# An Important Little Work on Biological Therapeutics

**Published:** 1909-10

**Authors:** 


### Reading Notices.

AN IMPORTANT LITTLE WORK ON BIOLOGICAL THERAPEUTICS.—In view of the near approach of the season when biological therapeutics will claim a considerable share of the attention of practitioners, reference may pertinently be made at this time to a unique and valuable contribution to the subject which has recently issued from the press of Messrs. Parke, Davis & Co. The publication consists of fifty-two pages, exclusive of the cover, and appears in brochure form. It is handsomely printed on white enamel paper of first quality and bears in colors a profusion of half-tone illustrations. The title is "Serums and Vaccines." A brief chapter on the origin and development of biological therapeutics, with an interjected hint as to what the opsonins may have in store for us, constitutes the introduction. Then follow chapters on serums—antidiphtheric, anti-tetanic, anti-streptococcic, anti-gonococcic, antitubercle and anti-venomous; on tuberculins; on vaccines, including the new bacterial vaccines which are exacting so much attention from the medical world; on organo-therapy, its development and some of the important products that are associated with it—"a tabulation,' in the language of the brochure itself, "of such creations of biologic pharmacy as are really utilizable in medicine." There are striking pictures of the company's home laboratories at Detroit, with numerous interior views; the research laboratory; the operating house and biological stables at Parksdale Farm (where the animals are cared for), with accompanying landscapes in nature's colors.

This little book, "Serums and Vaccines," is distinctly "worth while." If you haven't seen a copy, drop Parke, Davis & Co. a postal card at their home offices in Detroit, mentioning this journal, and get one. It is a safe guess that any physician who receives the brochure will read it admirably and with interest, filing it away thereafter for future reference.

